# Coinfections and comorbidities in African health systems: At the interface of infectious and noninfectious diseases

**DOI:** 10.1371/journal.pntd.0006711

**Published:** 2018-09-20

**Authors:** Derick Nii Mensah Osakunor, David Moinina Sengeh, Francisca Mutapi

**Affiliations:** 1 Centre for Infection, Immunity and Evolution, Institute of Immunology and Infection Research, University of Edinburgh, Ashworth Laboratories, Edinburgh, United Kingdom; 2 International Business Machines Research Africa, Johannesburg, South Africa; 3 National Institute for Health Research, Global Health Research Unit Tackling Infections to Benefit Africa, University of Edinburgh, Ashworth Laboratories, Edinburgh, United Kingdom; Universidade Federal de Minas Gerais, BRAZIL

## Abstract

There is a disease epidemiological transition occurring in Africa, with increasing incidence of noninfectious diseases, superimposed on a health system historically geared more toward the management of communicable diseases. The persistence and sometimes emergence of new pathogens allows for the occurrence of coinfections and comorbidities due to both infectious and noninfectious diseases. There is therefore a need to rethink and restructure African health systems to successfully address this transition. The historical focus of more health resources on infectious diseases requires revision. We hypothesise that the growing burden of noninfectious diseases may be linked directly and indirectly to or further exacerbated by the existence of neglected tropical diseases (NTDs) and other infectious diseases within the population. Herein, we discuss the health burden of coinfections and comorbidities and the challenges to implementing effective and sustainable healthcare in Africa. We also discuss how existing NTD and infectious disease intervention programs in Africa can be leveraged for noninfectious disease intervention. Furthermore, we explore the potential for new technologies—including artificial intelligence and multiplex approaches—for diagnosis and management of chronic diseases for improved health provision in Africa.

## Introduction

The top 10 diseases that account for the most disability-adjusted life years (DALYs) and cause of death in Africa include both infectious and noninfectious diseases, with the amount of DALYs contributed by noninfectious diseases almost catching up to those of infectious diseases [1]. What these data do not indicate is the level of comorbidity within the population, a reflection predominantly of the vertical management of diseases in African countries and a legacy of the historical focus on communicable diseases. In particular, when reporting causes of death, the contribution of comorbidities arising from infectious and noninfectious diseases is not reported. Population studies indicate that several tropical infectious diseases show common epidemiological patterns with age and share risk factors, including poor sanitation and lack of safe water [2]. Environmental and socioeconomic factors contribute to the coexistence of these pathogens in the same individual and cause concomitant morbidity [2].

**Fig 1:**
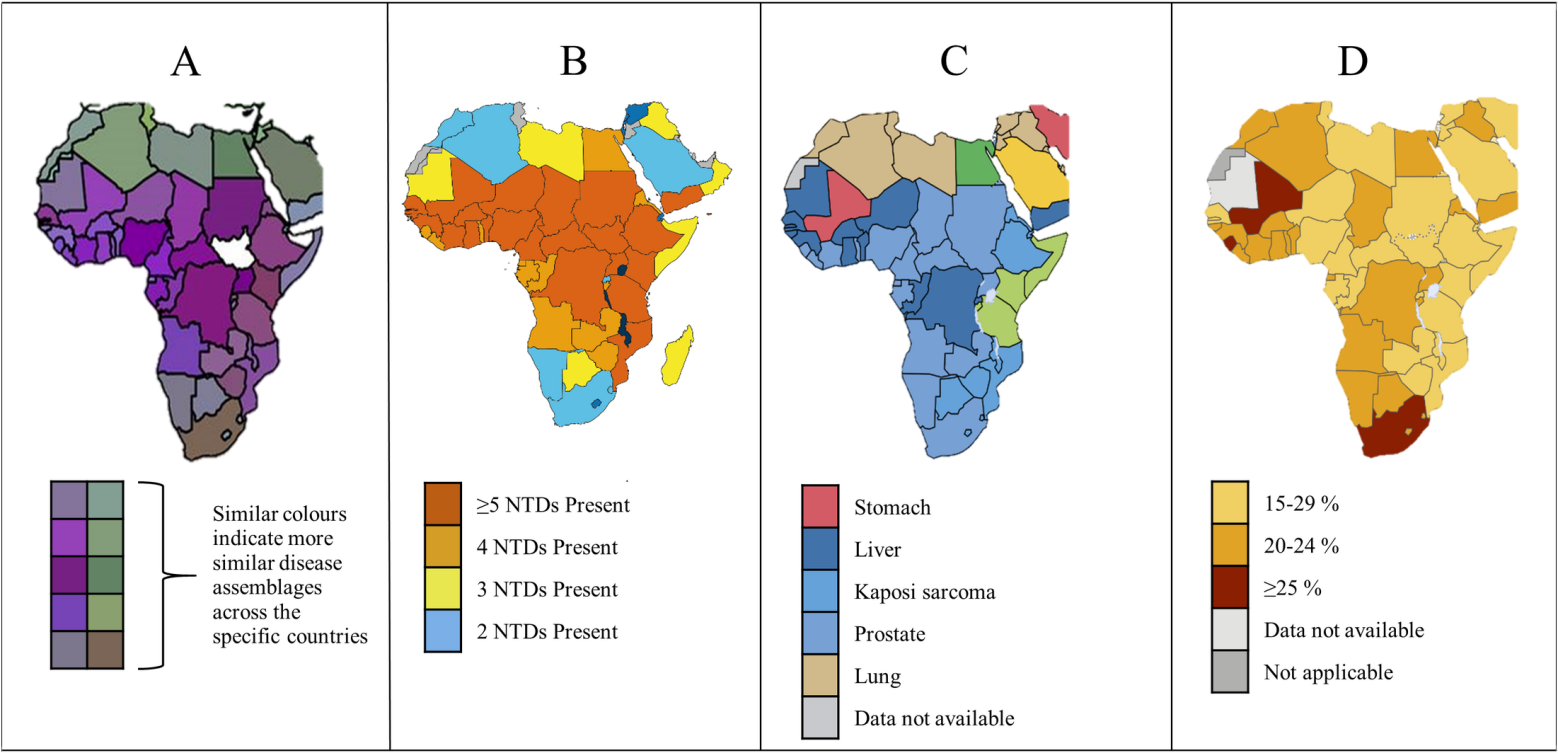
Adapted maps of Africa showing the overlap of neglected tropical diseases (NTDs), infectious, and noninfectious diseases. The figure shows (A) pathogeographic patterns of 187 global human infectious diseases [3], (B) patterns of the six most common neglected tropical diseases [4], (C) burden of the most frequently diagnosed cancer among males [10], and (D) probability of dying from the four main noninfectious diseases between the ages of 30 and 70 years [11]. Infectious diseases show distinct spatial patterns (A), which overlap with the most common neglected tropical diseases (B), commonly diagnosed cancers (C), and the mortality rates from major noninfectious diseases including cardiovascular diseases, cancer, chronic respiratory diseases, and diabetes (D).

Infectious disease co-occurrence exhibits distinct spatial patterns [3]. This co-occurrence, so-called pathogeographic patterns (Fig 1), observed in sub-Saharan Africa (SSA), overlaps with the distribution of neglected tropical diseases (NTDs) [4] and cancers, directly linked to infections (Fig 1). These NTDs include bacterial, parasitic, protozoal, and viral infections, as per the World Health Organisation (WHO) NTD list from the 10th meeting of the WHO Strategic and Technical Advisory Group for NTDs in 2017 (http://www.who.int/neglected_diseases/diseases/en/), with the most common NTDs being helminth parasites [4]. Helminths have been implicated in several noninfectious diseases including endomyocardial fibrosis [5], hypertension [6, 7], iron deficiency anaemia [8], and cancer [9].

**Fig 2:**
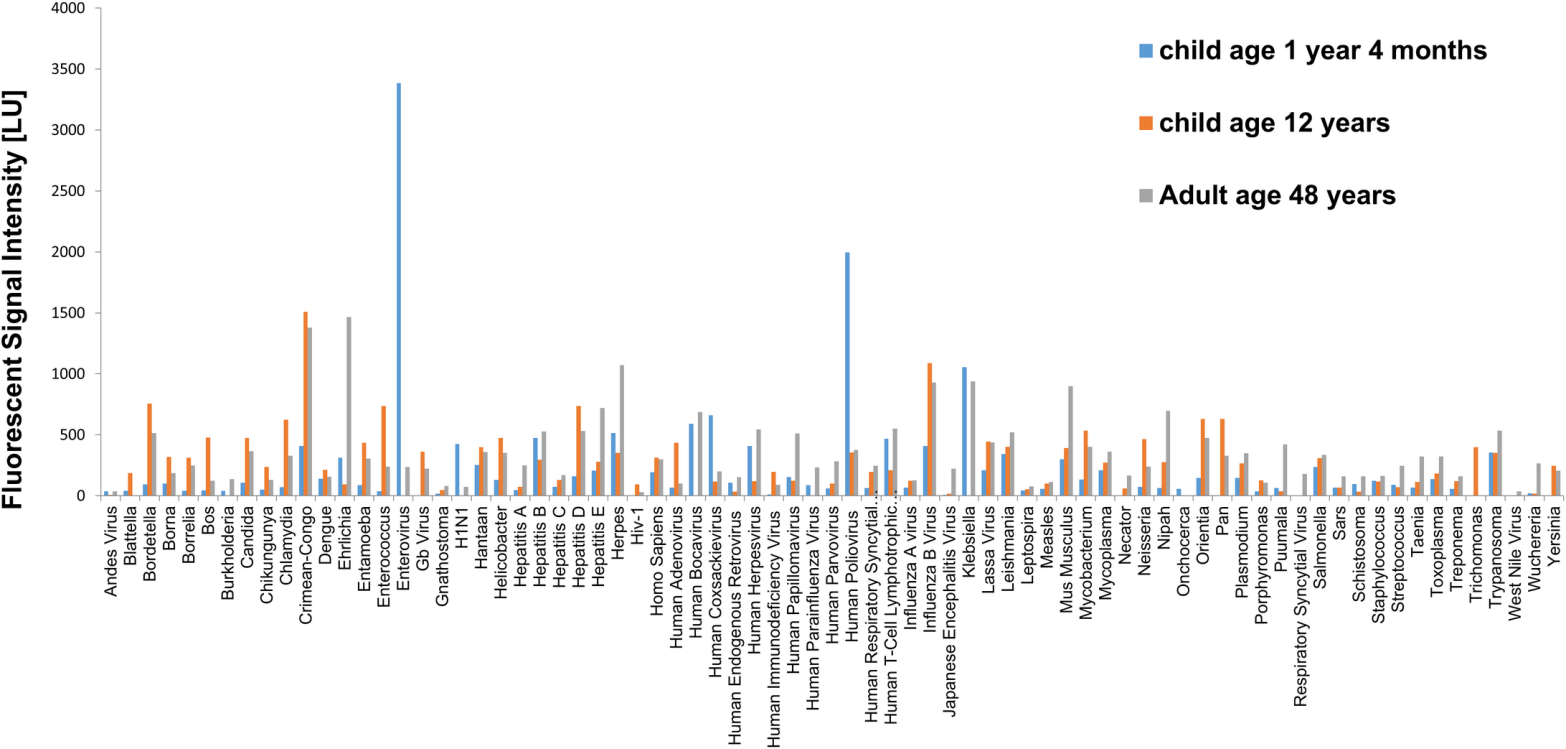
Host infectome analysis based on IgM reactivity to multiple infections in a Zimbabwean cohort. Results indicate variable responses to infections across all age groups. IgM, immunoglobulin (Ig) M.

In a recent pilot study, we evaluated a multiplex immunoglobulin (Ig) M and IgG antibody response fingerprinting platform for determining exposure history to pathogens using serum from a Zimbabwean population. Initial analysis showed evidence of recent exposure (IgM) to an array of infections (Fig 2). This platform also allowed the detection of responses to childhood vaccinations, as indicated by the high titres of responses against poliovirus in the child compared to the adolescent and adult. Although this exposure history requires further validation through other diagnostic methods, including parasitology and molecular approaches, it gives an indication of potential coinfections, as already described by others [3, 4].

In addition to diseases arising from infectious pathogens, there is also an increase in chronic noninfectious diseases, including high blood pressure, cardiovascular diseases, diabetes, and cancer. Direct and indirect interactions between infectious and noninfectious diseases have been poorly studied, particularly in African settings. For instance, there are now suggestions that mental illness may be an inflammatory disease [12, 13], but the sources of inflammation and their relative contribution to mental illness have yet to be determined. Aetiological and mechanistic experimental studies suggest that NTDs, including parasitic infections, may contribute to this inflammation [14]. For example, helminth infection during pregnancy has been suggested to impair neurocognitive development in infants [15], but mechanistic studies have yet to be conducted. Apart from well-known infections, such as human immunodeficiency virus (HIV) and human papilloma virus (HPV) that are risk factors for some cancers [16, 17], there is increasing evidence that inflammation from infectious pathogens contributes to the aetiology of diabetes and coronary artery disease [18, 19].

With increasing coinfections and comorbidities, there is a need to investigate aetiological links between these two groups of diseases (infectious and noninfectious), and to invest in horizontal health systems approaches and training of healthcare workers to manage multiple and chronic conditions. In this review, we hypothesise that the growing burden of noninfectious diseases may be linked directly/indirectly, or further compounded by the existence of NTDs and other infectious diseases. We explore the challenges/barriers to implementing effective and consistent healthcare in SSA in the face of the observed disease trends. We discuss how existing NTDs and other infectious disease intervention programmes and infrastructure can be leveraged for noninfectious disease intervention, diagnosis, and long-term management of diseases, for improved health provision in Africa.

## Methodology

A literature review was conducted using electronic databases, including Pubmed/Medline, Google Scholar, and WHO (http://www.who.int). For the entire review, we searched for research articles with keywords relevant to each section of the review. We analysed all articles published and included those relevant to the scope of this review. A systematic review of literature (PubMed) was done to determine the impact of coinfections in Africa. Search terms for the systematic review were (a) [(Co-infection* OR Coinfection*) AND (Co-morbid* OR Comorbid*) AND (Africa) AND (Health impact*)] (b) [(Co-infection* OR Coinfection*) AND (Co-morbid* OR Comorbid*) AND (Africa) AND (Health impact*) AND Helminth*)]. Selection criteria included human studies, original articles, studies that relate coinfection or comorbidity to a secondary health impact, and articles published in the last 10 years as at January 2018.

## Health impact of coinfections

The outcome of coinfections can be asymptomatic, symptomatic, and sometimes fatal. While there are studies—predominantly in experimental models—suggesting the health benefits of infection, e.g., with helminth infections as described through the hygiene hypothesis [20], there are few studies from human populations. These experimental studies may be informative at the mechanistic level, but their phenotypic and thus clinical relevance in humans requires careful and well-designed studies. Animal models of natural infection bridge the gap between experimental and human studies, and these indicate that coinfections can influence population-level disease and mortality patterns, which ultimately influence interventions. For example, a study in African cattle showed that concurrent infection of *Theileria parva* with less pathogenic species of *Theileria* resulted in a reduction in *T*. *parva*-associated mortality [21]. Nonetheless, parasite coinfections in a cattle study showed antagonistic effects that compromised the health of cattle [22].

In human populations, detrimental effects of coinfections have been reported. Polyparasitic infections in Africa have been associated with a higher tendency for wasting, splenomegaly, and anaemia [23]. As shown in Table 1, a systematic review of literature on the health impacts of coinfections in the SSA region suggests the apparent lack of adequate research evidence on the subject matter. The impact of coinfections on health will become more evident as more holistic approaches are taken to studying the health of the host rather than focusing on just pairs of parasite–host relationships or on the interaction and impact of two infections (predominantly HIV and another disease) as has been the predominant practice.

**Table 1:** Summary of publications on health impacts of coinfections in Africa within the last 10 years.

Year	Source	Disease dynamics	Health impacts
2007	Hoffmann and Thio 2007 [24]	Hepatitis B virus–HIV	Liver enzyme alterations, reducing antiretroviral tolerance and increasing its toxic effects. Blunt immune recovery from antiretroviral therapy.
2009	Hadley and Naude 2009 [25]	HIV–Tuberculosis–Malignant tumours	Increased mortality.
	Degarege, Animut et al., 2009 [26]	Malaria–Soil-transmitted helminths	Impact on malaria severity, although small.
2010	lsa, Gwamzhi et al., 2010 [27]	Hepatitis B and C viruses–HIV/AIDS	Impact on causing hepatotoxicity.
	Sangweme, Midzi et al., 2010 [28]	Schistosomiasis–Malaria	Higher peripheral blood malaria parasite density, promoting transmission.
	Modjarrad and Vermund 2010 [29]	HIV–Tuberculosis–Syphilis	Tuberculosis and syphilis may increase HIV viral load, increasing disease progression.
2012	Ntusi, Badri et al., 2012 [30]	*Acinetobacter baumannii*–HIV/AIDS	Increased mortality.
	Faurholt-Jepsen, Range et al., 2012 [31]	Tuberculosis–Diabetes	Poor treatment outcomes including delayed recovery of body mass and haemoglobin levels, hence poor recovery from disease.
	Webb, Barrett et al., 2012 [32]	Chronic myeloid leukaemia–HIV	Poor cytogenic response to leukaemia treatment.
	van den Bogaart, Berkhout et al., 2012 [33]	Visceral leishmaniasis–Malaria	Early detection results in good prognosis, but patients stand a high risk of severe symptoms of leishmaniasis.
2013	Ladep, Agbaji et al., 2013 [34]	Hepatitis B virus–HIV	Reduced survival. With the appropriate treatment Tenofovir, this impact may be annulled
	Taye, Alemayehu et al., 2013 [35]	Podoconiosis–Soil-transmitted helminths	Increased blood losses/anaemia.
2014	Baldassarre, Mdodo et al., 2014 [36]	HIV/AIDS–Cryptococcal meningitis	Increased mortality.
	Knight, Muloiwa et al., 2014 [37]	HIV–Stevens Johnson syndrome–Toxic epidermal necrolysis	Increased risk of systemic bacterial infection and mortality.
	Biraro, Egesa et al., 2014 [38]	Helminths, malaria, or HIV coinfection in household contacts of Tuberculosis patients	No evidence of increased risk to latent Tuberculosis. Th1 cytokine responses in those with prior BCG vaccination was reduced.
	Degarege, Animut et al., 2014 [39]	Malaria–Helminths	Undernutrition; severity is comparable to those with single infections.
2015	Umanah, Ncayiyana et al., 2015 [40]	HIV–Tuberculosis	Treatment failures and increased mortality.
2017	Morawski, Yunus et al., 2017 [41]	HIV–Hookworm	Decreased CD4^+^ T cell counts during antiretroviral therapy.

Systematic review of literature (PubMed); electronic search terms were (a) [(Co-infection* OR Coinfection*) AND (Co-morbid* OR Comorbid*) AND (Africa) AND (Health impact*)] (b) [(Co-infection* OR Coinfection*) AND (Co-morbid* OR Comorbid*) AND (Africa) AND (Health impact*) AND Helminth*)]. Selection criteria: human studies, original articles, and studies that relate coinfection or comorbidity to a secondary health impact published in the last 10 years.

**Abbreviations:** AIDS, acquired immune deficiency syndrome; BCG, Bacillus Calmette–Guérin; CD4^+^, cluster of differentiation 4.

## Epidemiology in transition

### From acute/episodic diseases to chronic conditions

In addition to communicable diseases, there is an increasing burden of noninfectious diseases such as hypertension, stroke, cancer, and diabetes in the SSA region. This concurrent health challenge is compounded by the lack of marked progress in the control of infection and malnutrition, if at all [42, 43]. The insurgence of noninfectious diseases is a “time bomb” for Africa, with the region expected to record the world’s largest increase in noninfectious disease deaths by 2030 [44]. Already, countries in northern and southern Africa account for more than three quarters and close to a half of all deaths to noninfectious diseases, respectively [45].

HIV is now a chronic infection; increased access to antiretroviral therapy (ART) has substantially improved health and reduced the risk of HIV transmission, increasing the life expectancy of HIV patients to one close to that of uninfected populations [46]. Thus, there is an increasing number of over 50-year-old patients living with chronic HIV, and the impact of HIV and ageing on the acquisition of noninfectious diseases like diabetes become key [46], requiring long-term management and care.

Lymphatic filariasis and onchocerciasis pose a serious public health problem in Africa, causing long-term chronic infection with permanent and long-term disability [47]. In human filariasis infections, coinfections with other infectious diseases is common and can affect protective immune responses for infections like malaria and tuberculosis (TB) [48]. Chronic long-term management of filarial infections thus become a very important component of healthcare. This is crucial, especially to prevent secondary infections that may worsen late-stage diseases.

**Fig 3:**
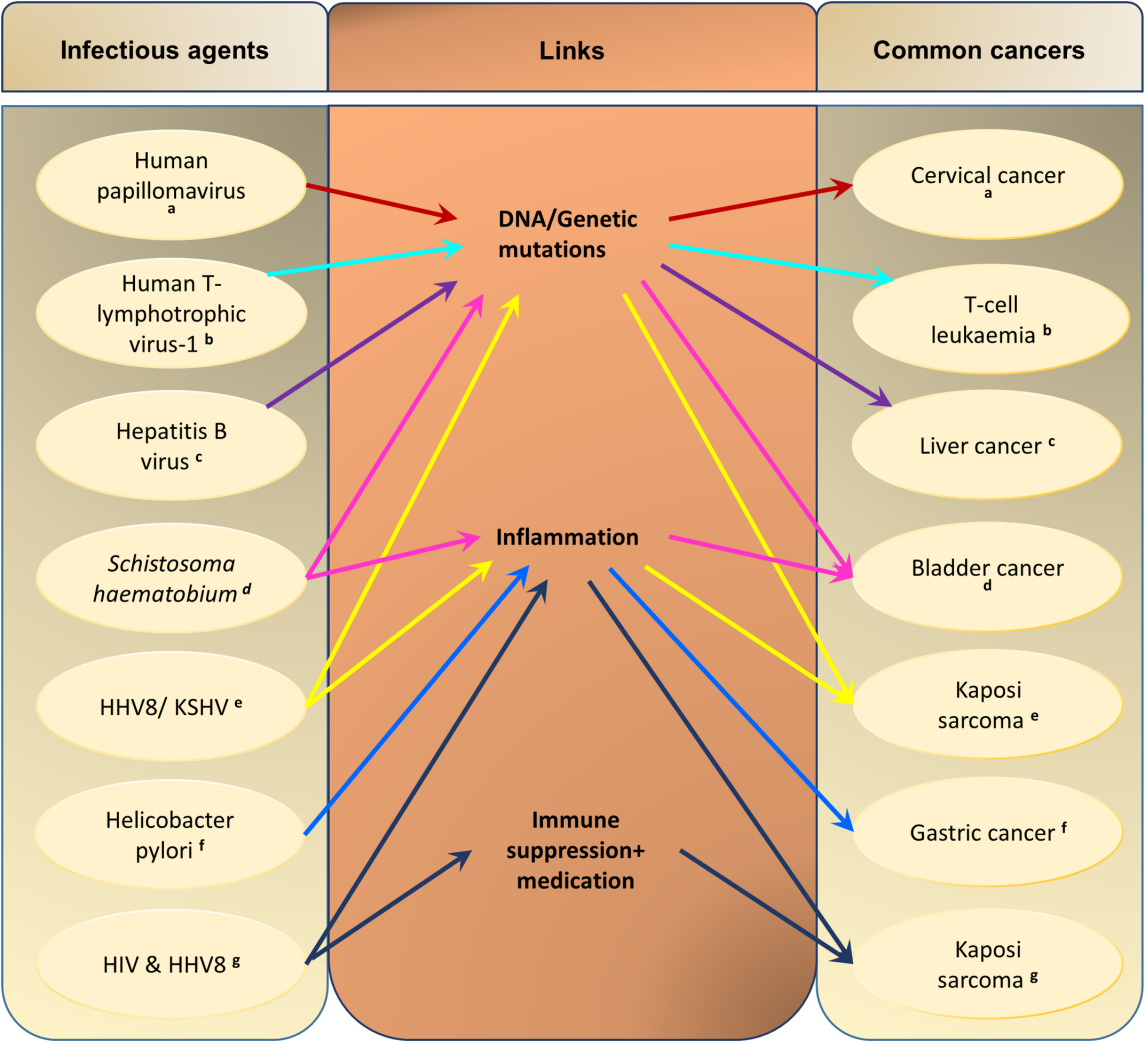
Summary of infections and the types of cancers they cause, via direct or indirect links. Each coloured line/alphabet represents a pathological pattern. Information adapted from ^a^Crosbie, Einstein, and colleagues, 2013 [16]; ^b^Ahmadi Ghezeldasht, Shirdel, and colleagues, 2013 [55]; ^c^Marra, Sordelli, and colleagues, 2011 [56]; ^d^Mostafa, Sheweita, and colleagues, 1999 [57]; ^e^Dittmer and Damania, 2016 [58]; ^f^Polk and Peek, 2010 [59]; and ^g^Bower, Nelson, and colleagues, 2005 [17]. HHV8, human herpes virus 8; KSHV, Kaposi sarcoma-associated herpesvirus; HIV, human immunodeficiency virus.

While there are many risk factors associated with the growing number of cancers in Africa, infectious diseases play a significant role (Fig 3). About a third of new cancers in Africa are due to viral, bacterial, or parasitic infections [49]. The implication of this increasing comorbidity of cancer and infectious diseases in Africa means that disease screening, diagnosis, treatment, and care need to be revised to determine potential multiple interventions.

Developing countries bear over 80% of the global cardiovascular disease burden [50]. At the same time, although previously rare, diabetes has emerged as an important noninfectious disease in SSA [51]. Such metabolic diseases are currently affecting individuals at a much younger age than when compared to developed countries [52]. While individualised approaches to preventing nutritional and metabolic disease can be effective in developed countries, they are not affordable or feasible for the poorer population in low-income countries. Therefore, societal approaches such as those that have been used in public health educational/awareness campaigns for infectious diseases, notably HIV, will have to be implemented.

The socioeconomic and cultural environment around this current epidemiologic situation in SSA differs from that in most Western countries. In SSA, noninfectious diseases were not anticipated, were accompanied by cultural misconceptions, and have historically received less attention and health budget allocation compared to communicable diseases [53]. Chronic diseases will require long-term management, incurring a cost for both the individual and an already overburdened healthcare system. However, healthcare systems in SSA are designed to provide more acute care, and many are ill-equipped to provide long-term care for chronic conditions, as exemplified in Tanzania [54].

### Problems arising from coinfection and comorbidity

#### The healthcare system

Despite some differences among health systems across Africa, these exhibit some similar structural and organisational formats. Table 2 summarises the general nature and challenges associated with national health systems across Africa by using model countries in different regions of the continent.

**Table 2:** Health systems in Africa: Structure and challenges.

Region	Model country	System structure	Challenges	Source
Anglophone	Tanzania	Bottom–up approach. Village health services for remote areas at level 1. Level 2 consists of dispensary services for localities with larger populations. Level 3 offers services to even larger populations, up to 50,000 people.	Lack of access for the poor due to the copayment system, insurance requirements, and the insurgence of private physician practices. Absenteeism, low morale, inadequate qualified work force, lack of equipment and supplies. Centralisation at the high level of care.	[63, 64]
	Kenya	Well organised and pyramidal, with dispensaries, health centres, subdistrict hospitals/private clinics, provincial and national hospitals.	Recurrent strikes by doctors, problems with financing health systems, high cost of health services, HIV/AIDS and malaria alone consumes the greatest part of resources.	[65]
	Uganda	Village health teams and community medicine distributors at level 1. Higher up is the health centre II in parishes, health centre III in sub-country, health centre IV, the regional referral hospitals, and three national referral and teaching hospitals.	Village volunteers can be unreliable, lower levels are quick to refer cases. Inadequate infrastructure, inequity in health services, lack of sustenance, low remuneration for staff, paucity of specialised physicians, poor training, high rates of staff layoffs. Poor data collection and utilisation.	[66]
Francophone	Cote d’Ivoire	Follows the 1996 health system organisation with three-tier pyramidal structure. Level 1: health, urban medical, school and university health centres. Level 2: general, regional and specialised hospitals. Level 3: specialised health institutes.	Low level of qualified personnel (one doctor per 10,000). High cost of universal healthcare led to its abandonment, hence lots of out of pocket care.	[63]
	Senegal	Similar structure to that of Cote d’Ivoire. Pyramidal with three levels. Central level: Ministry of Health. Regional level: local health systems. Peripheral level: health districts.	Disparities in distribution of facilities across the country. Sustained by government budget and relies a lot on donor support. Inadequate workforce, inadequate training, poor infrastructure and communication machinery. Social and religious barriers with disparities in quality of care.	[63]
Lusophone	Angola	Has three levels. Primary level: referral health centres or district hospitals, health posts. Secondary care: specialised facilities and general hospitals. Tertiary care: specialised health facilities and central hospitals	Lack of proper remunerations, inadequate allocation of resources by leadership, lack of decentralisation, persistent shortage of essential drugs, lack of data collection and availability.	[67]
	Mozambique	Has four levels. Primary level: health posts (the least equipped) and health centres. Secondary level: rural hospitals and urban hospitals. Tertiary level: five general and seven provincial and district hospitals. Quaternary level: three central hospitals.	Shortage of qualified staff to brain drain, and the system has some of the lowest salaries in Africa. Over reliance on foreign donor support makes it unsustainable. Poor infrastructure and absence of diagnostic tools. Inequitable distribution of health facilities.	[68]
Hispanophone	Equatorial Guinea	Similar structure to that of other countries with a national Ministry of health, Tertiary, Secondary, and Primary healthcare facilities.	Poor leadership and governance, low health financing (93.5% of health cost is out of pocket). Poor service delivery, lack of skilled physicians, and poor management of medical resources. Lack of available health data countrywide.	[69]

The coexistence of multiple infectious and noninfectious diseases, characterised by multiple comorbidities, presents unique problems for healthcare delivery in SSA. From the simulated global economic output losses of US$47 trillion from noninfectious diseases over the next few decades [60], low- and middle-income countries are unlikely to be equipped to bear most of this burden. Lessons from chronic HIV management suggest that identifying infected individuals, creating access to therapy, and addressing the multiple complications associated with long-term care requires a well-resourced healthcare system [61]. African health systems are geared toward a more episodic regime of healthcare, without capacity to absorb more patients into chronic disease care in an efficient, affordable, and sustainable manner [54]. Multiplex disease management models, differential diagnostic ability, and proper interventions are essential for long-term patient care. The success of HIV management, including the UNAIDS HIV 90:90:90 care continuum [62], relies on adherence to prescribed medicines, long-term follow up of patients, self-management, and behavioural change by patients.

Health systems in Africa need to be strengthened to improve effectiveness and efficiency across both rural and urban areas, reducing resource wastage, tailoring the training of healthcare providers to the needs of the specific population, along with proper compensation for healthcare providers. Currently, the health system across Africa follows different structural and funding models (see Table 2) and lessons can be learnt from the different models to improve on service delivery and accessibility for comorbidities.

### Diagnosis

#### What is the patient suffering from?

The existing infrastructure in most parts of SSA is not equipped for differential diagnosis. Appropriate basic diagnostic tests to support clinical symptoms may be lacking. For example, in Tanzania, less than 50% of patients with severe malaria (based on WHO clinical criteria) were laboratory confirmed [70], and in Ghana, 40% of such patients were confirmed to have bacterial sepsis and not malaria [71]. Conditions like anaemia, which are common in areas coendemic for different pathogens, still have no simple point-of-care diagnostic tests available. In some cases, despite availability of the technology, conditions are still not diagnosed, e.g., in 15% of Kenyan children with a clinical history of anaemia or malaria, haemoglobin levels were not measured [72]. Where there are diagnostic tests available, their utility may be compromised by the lack of a reference standard test, as occurs in childhood peritoneal tuberculosis (CPTB). In a modelling analysis of five different methods, including the sensitive mycobacterial cultures, tests failed to detect almost 40% of CPTB [73]. In addition, there are reports of challenges with quality control and reproducibility, with very few or nonexistent national laboratory guidelines [74]. Most available diagnostic tests are usually validated in Western populations, without guidelines for use in different populations where a different disease ecology exists. A study in a helminth-infected African population showed that routine allergy diagnostics are impaired by IgE antibodies to the carbohydrate epitope galactose-α-Gal, induced by the parasite infection [75]. Hence, some diagnostic tests used in the SSA region may be failing due to not having been developed/optimised for use in polyparasitic individuals or those presenting with comorbidities.

Lack of centralisation of services impacts healthcare delivery, e.g., in HIV management, nonprofit and commercial organisations are operating specialised independent laboratories [74], which leads to an efficient but exclusive vertical system for HIV management in populations affected by other infectious and noninfectious conditions. Indeed, the absence of evidence-based medicine contributes to poor patient outcomes, misdiagnosis in favour of more common illnesses, delayed treatment, and significant morbidity and mortality.

#### Patient engagement in healthcare

The importance of the role of the patient in healthcare decisions is increasingly and internationally being recognised, particularly in interventions integrating behavioural change [76]. Central to patient engagement is communication, i.e., communicating the diagnostic procedure and results, followed by interventions accessible to the patient. In African health systems, context-appropriate communication of the diagnosis to the patient is challenged by poor education and knowledge of the disease process. A study conducted in South Africa showed that within the African cultural context, most patients viewed the definite diagnosis as having been bewitched, associated it with poor prognosis, and barely understood their diagnosis. On the other hand, health workers expressed concern of inadequate training and lack of competence in communicating diagnosis [77]. In rural Cameroon, most patients tend to disagree with the diagnosis, depending on how well they understood explanations given by the provider. Practitioners often do not give appropriate explanations, do not support patients to express their opinions, and tend to show signs of disapproval when patients do [78]. The ability to communicate diagnosis and, in effect, treatment options becomes more important in the context of coinfections and comorbidities. These highlight important obstacles to appropriate patient care and the need to include proper patient–provider communication as part of healthcare delivery in the face coinfections and comorbidities. Knowledge, attitude, and practice (KAP) studies, including those on both infectious [79] and noninfectious diseases [80], indicate that poor knowledge is associated with practices that increase risk of disease or poor disease management.

#### What has the patient died of?

In addition to establishing a final diagnosis, autopsies relate the cause of death to associated pathologies that may be present, thereby establishing an interaction [81]. This is important in helping health experts find and track outbreaks, routine diseases and hazards, and helps family members be aware of the genetic risk of diseases. In most parts of SSA, issuing a death certificate is not mandatory and full autopsies are rare due to resource constraints and unwillingness of families to have an autopsy performed [82]. This leads to imprecise approaches to determining the cause of death. A major constraint on global health and development is the absence of mortality patterns due to specific diseases, raising questions on how representative available data are, in relation to populations that go uncounted for. Verbal autopsy is used as an alternative low-cost approach to determine cause of death, and WHO has developed international standards for verbal autopsy, revising its use with automated models [83]. This can be improved by combining verbal autopsies with minimally invasive autopsies (MIAs), an initiative endorsed by funding agencies such as the Bill & Melinda Gates Foundation (BMGF) [84]. Already, reports from Mozambique show significant agreement of MIA with full autopsies [85]. In most of these areas, where MIA is likely to be of benefit, infrastructure such as advanced radiology may not be available, and if at all, it may be expensive. Others have suggested that MIA protocols dependent on needle sampling be used in low- and middle-income countries, although its suitability has yet to be determined [86]. Data from this MIA–verbal autopsy system could be informative for improving future verbal autopsy standards and improving viability and cost of large-scale cause-of-death assignments within SSA.

### Interventions

#### What is the desired outcome?

Diagnosis does not mean cure; therefore, advances in diagnostics must be matched with advances in interventions. Interventions must be informed by knowing what the desired outcome is and what tools are required or available to achieve this. For instance, there is need for a definition of what constitutes a healthy or sick African and what constitutes a healthy or weak immune system amidst all the coexisting infections and morbidities. For example, in a Ugandan healthy population, significant disparity has been described in absolute laboratory values when compared to populations outside SSA, suggesting the necessity to develop specific ranges for the African population [87]. Such heterogeneity is important for contextualising interventions, e.g., initiation of antiretroviral therapy among HIV patients is informed by CD4 cell counts and any immune reconstitution interventions.

#### What is the most appropriate drug to use?

Administering treatment in populations affected by coinfections and requiring chronic long-term management requires sufficient knowledge of the type and species of infection, drug–drug interactions within specified populations to inform dosage, and the impact on drug resistance and treatment efficacy. For instance, experience from malaria intervention programmes shows that treatment regimen depends on the target species [88], and our recent studies indicated that repeated treatment was required in multi-*Plasmodium* species malaria-infected individuals when compared to individuals with single species infection (Amanfo and colleagues, in prep). Due to high prevalence of some conditions, clinicians may favour clinical diagnosis against laboratory evidence, treating symptoms instead of causes. For example, in malaria endemic areas, fever may not always be malaria [74], and in the advent of rapid diagnostic tests (RDTs), even in hard to reach areas, majority of these tests may come out negative; in 2014, about 142 million suspected cases of malaria tested negative worldwide [89]. With similar tests lacking for other diseases that cause fever, health workers are left in a dilemma and with nothing to offer. In 2016, a high proportion of febrile children in Africa did not receive medical attention due to poor access to healthcare and lack of awareness among caregivers [89]. Building a stronger health system to deal with such challenges is recommended [90]. Ideally, in cases of patients receiving multiple drugs for multiple conditions, drug–drug interactions need to be considered and managed to maximise efficacy while reducing toxicity. For example, in the administration of the antihelminthic Praziquantel along with Albendazole in multiparasitic interventions, the routine coadministration of both drugs may affect the total exposure of Albendazole [91].

Defining the impact of heritable traits on pharmacology and toxicology in African populations is essential for targeted interventions. For example, cytochrome P450 variants impact drug metabolism [92]. The application of pharmacogenetics can allow prediction of drug efficacy or failure in patients before a drug is deployed, saving time and cost from trial and error prescriptions [93] and may indirectly reduce the development of resistance [94]. Although this requires significant investment, it is clear that near-personalised management of HIV patients already occurring in Africa has already set the precedent.

## Potential solutions

### Leveraging existing platforms within health systems for disease control

Africa can leverage the successes of infectious disease control programmes to address the increasing burden of noninfectious and chronic diseases. These must encompass innovations that include both prevention and healthcare delivery.

### Operational approaches

In many African countries, routine healthcare in the community is delivered through Community Health Workers/Volunteers (CHW). These CHWs are helping efforts to achieve universal healthcare at a low cost per person served. They have been trained to support chronic care and long-term interventions such as supporting community engagement and education, mass drug campaigns for NTD interventions, and maintaining compliance to HIV and tuberculosis treatment. For example, in a population with a high prevalence of HIV, Chibanda and colleagues initiated a low-cost “friendship bench” intervention, locally adapted from problem-solving therapy, to manage mental disorders [95]. In context, such interventions can deliver a successful, practical, yet culturally accepted treatment programme for long-term management of cases [96], with indirect benefits for compliance to treatment for HIV. In SSA, programmes such as these are rewarding for CHWs and can be sustained over long periods at low costs, hence applicable in the context of available poor health systems. Lessons learnt from HIV control in Malawi are being applied through integrating screening for hypertension into HIV care [97], and in Ghana, decentralised community-based hypertension care has been adapted from HIV management [98].

Control of infectious diseases can also be integrated for greater health impact as exemplified by antihelminthic treatment. Schistosomiasis has been linked to malaria infection in children [99], and schistosomiasis treatment in areas where malaria is coendemic has been shown to reduce malaria transmission [100]. Anthelimintic treatment, in addition to killing the parasites, has been shown to restore neurocognitive performance in school children [101, 102].

Of critical importance is the monitoring and evaluation of any changes within health systems, including integrating health service delivery into existing platforms. For example, what impact does the introduction of a new vaccine have on the health system? Does the expanded programme on immunization (EPI) system in affected countries adjust to cope with such impacts or does this create strains in the system?

### Optimising current interventions

Current interventions can be optimised to prevent multiple disease conditions. For example, coadministration of the childhood vaccinations against influenza type B, whooping cough, tetanus, hepatitis B, and diphtheria as a single formulation (Pentavalent) [103] increases compliance. There are already indications that some current vaccines can have broad spectrum effects; the Bacillus Calmette–Guérin (BCG) vaccine can boost the immune system to resist multiple infections [104].

Promotion of already existing measures such as extended breastfeeding programmes has a positive impact on childhood disease and pathology. Breastmilk can contribute to bioactive factors of the innate immune system as well as enhance the protective ability of the gastrointestinal tract [105].

### Integrating other platforms into health systems for control

#### Probiotics and disease control

The utility of probiotic therapy in maternal and child health presents great potential for disease prevention and management, and its role has been extensively reviewed [106]. Experimental studies have shown that intranasal probiotics of *Lactobacillus* strains stimulate immune responses in the respiratory tract, offering protection from viral (H1N1) infection [107, 108]. Probiotics have been used to deliver antigens or adjuvants directly to the “unfriendly” gastrointestinal tract in HIV vaccine development [109] and as a potential cryopreservative and immunomodulator of mucosal immune response in Hepatitis B vaccines [110]. Despite existing evidence on the role of probiotics to enhance vaccine-specific immunity, there is a need for rigorous longitudinal mechanistic and efficacy studies in paediatrics for different vaccines.

#### Technology and artificial intelligence

The use of artificial intelligence (AI), a specialised branch of computer science that deals with the ability of computers to perceive their environment and make decisions to maximise the chances of success of an event or goal, in African health systems is still limited but presents great potential. AI solutions can be used for decision support/validation, multiple-disease screening and diagnosis, including the use of genomic data and treatment optimisation within resource-constrained environments. A recent report presented a solution that integrates and analyses data across various sources, including disease incidence for clinical and operational decision support at the district level in Sierra Leone [111]. Disease screening, which involves reading images, can be fully or partially automated using advances in computer vision and AI algorithms, coupled with the widespread availability of cell phones in Africa to enhance human expert capacity [112]. Network analyses can be used to establish links between diseases, ultimately informing treatment plans at the individual and population level [113]. Improving the rate of uptake and integrating AI platforms to electronic health records will improve individual healthcare as well as strengthen the African health system [114].

Mobile devices have become very popular in Africa and present great potential for improved healthcare delivery. An example is the mTRAC mobile health system being used in Uganda to report available stocks of medicines and the mHealth in Kenya to better understand the supply chain of medicines [115]. These will ensure that medicines reach patients who need it the most.

## Where to go from here

Overall, we are in an era in which there is long-term survival and management of chronic conditions. Some health systems have resources for chronic long-term care and support groups are available in some countries to empower and promote healthy lifestyles for patients living with such conditions; an example is Diabetes South Africa. The challenge is thus to equip current health systems to shift from episodic interventions for acute care and be resourced for chronic care and to make existing support groups and systems readily available to the poorest and illiterate groups of affected patients.

Adequate training should be available to next generation scientists and health workers to build local health, research, and development capabilities. Ongoing programmes include the Human Health and Heredity in Africa (H3Africa), aimed at training local scientists from Africa to develop treatments for conditions including infectious (e.g., tuberculosis and malaria) and noninfectious diseases (e.g., cardiovascular disease) [115]. Guidelines, drugs, and appropriate monitoring equipment also need to be in place, accessible but affordable to all and tailored to different levels of care [51].

The role of education through interactive media and mobile devices cannot be overemphasised. There have been calls for funding agencies, the media, and health institutions in Africa to be partisan in health knowledge generation and application beyond publication in scientific journals [116]. Development partners have promoted innovative ways of delivering HIV and reproductive health education to young people; for example, the MTV Shuga show (http://www.mtvshuga.com/). The use of mobile phones is on the rise in Africa and present enormous potential for mobile health beyond direct patient care [117]. The mobile messaging platform WhatsApp has been demonstrated to be effective in enhancing the supervision of CHWs and creating innovative forms of community-based digitally supported professional development with minimal training [118]. This will go a long way to strengthen the formal healthcare system and enhance the role of CHWs in hard-to-reach areas. The photo sharing platform Instagram has been used by WHO, CDC, and others to broadcast public health messages for education and for sensitisation during public health crises [119].

A marked difference in healthcare can also be achieved through public–private partnerships. Ongoing initiatives like the Foundation for Chronic Disease Management (FCDM) by Novartis, in collaboration with International Business Machines (IBM) and Vodacom, is doing a great job by linking public health workers to those in the private sector to deliver quality but affordable healthcare to homes [115].

By conducting more target-oriented research in multiple disease systems, more realistic interventions will be achieved quickly for coinfections and comorbidities. To do this, researchers must shift toward collaborative and multidisciplinary studies, which can aid in understanding disease interactions and their impact on overall health. Funders should be more willing to support such studies and be willing to fund riskier innovative research programmes with potential to benefit the health and wellbeing of millions of people. In addition, governing bodies and policy makers should be willing to incorporate findings from such studies and to prioritize both infectious and noninfectious diseases management.

**Key learning points**

- There is an epidemiological transition in sub-Saharan Africa (SSA), with the insurgence of coinfection and comorbidities from both infectious/neglected tropical diseases (NTDs) and noninfectious diseases.
- Health systems in SSA are ill equipped to deal with this in terms of diagnosis, intervention, and long-term care.
- Basic scientific research in SSA must be target oriented, collaborative, and on multiple disease systems (i.e., horizontal approach) to enhance our understanding of disease interactions and their impact on overall health and to improve implementation.
- Current success stories or interventions in the management of infectious and NTDs in SSA can be leveraged for noninfectious diseases, addressing coinfections and comorbidities.

**Top five papers**

1. Woolhouse ME, Thumbi SM, Jennings A, Chase-Topping M, Callaby R, Kiara H, et al. Co-infections determine patterns of mortality in a population exposed to parasite infection. Sci Adv. 2015;1(2):e1400026. doi: 10.1126/sciadv.1400026. PubMed PMID: 26601143; PubMed Central PMCID: PMCPMC4643819.
2. Podoconiosis and soil-transmitted helminths (STHs): double burden of neglected tropical diseases in Wolaita zone, rural Southern Ethiopia. PLoS Negl Trop Dis. 2013;7(3):e2128. Epub 2013/03/22. doi: 10.1371/journal.pntd.0002128. PubMed PMID: 23516659; PubMed Central PMCID: PMCPMC3597475.
3. Bryan L, Conway M, Keesmaat T, McKenna S, Richardson B. Strengthening sub-Saharan Africa’s health systems: a practical approach. McKinsey Quarterly. 2010.
4. Nabyonga J, Orem J. From Knowledge to Policy: Lessons from Africa. Sci Transl Med. 2014;6(240). doi: ARTN 240ed13 10.1126/scitranslmed.3008852. PubMed PMID: WOS:000337909300006.
5. Chibanda D, Mesu P, Kajawu L, Cowan F, Araya R, Abas MA. Problem-solving therapy for depression and common mental disorders in Zimbabwe: piloting a task-shifting primary mental health care intervention in a population with a high prevalence of people living with HIV. BMC Public Health. 2011;11:828. doi: 10.1186/1471-2458-11-828.
